# Benzoin Resin: An Overview on Its Production Process, Phytochemistry, Traditional Use and Quality Control

**DOI:** 10.3390/plants12101976

**Published:** 2023-05-14

**Authors:** Qingqin He, Yuanyuan Sun, Xiqin Chen, Jian Feng, Yangyang Liu

**Affiliations:** 1 Institute of Medicinal Plant Development, Chinese Academy of Medical Sciences and Peking Union Medical College, Beijing 100193, China; heqqin.77@gmail.com (Q.H.); sy232231225@gmail.com (Y.S.); chenxiqin6@gmail.com (X.C.); 2 Hainan Branch of the Institute of Medicinal Plant Development, Chinese Academy of Medical Sciences and Peking Union Medical College, Haikou 570311, China; jianfenghannan@gmail.com

**Keywords:** benzoin, production, phytochemistry, traditional use, quality control, review

## Abstract

Benzoin is a pathologic exudation produced by plants of the family *Styrax*. It is secreted by traumatic resin ducts after injury, which are derived from parenchymal cells in secondary xylem by schizolysigeny. Some 63 chemical constituents have been isolated and identified from this resin, including balsamic acid esters, lignans and terpenoids. It has a long history of applications, including as incense along with olibanum, a flavor enhancer in the food industry, materials in the daily chemistry industry as well as therapeutic uses. Up to now, high-performance liquid chromatography (HPLC) and gas chromatography mass spectrometry (GC-MS) have been widely used in qualitative and quantitative analysis of benzoin. Other technologies, including near-infrared reflectance spectroscopy (NIR), proton transfer reaction time-of-flight mass spectrometry (PTR-ToF-MS) and Fourier-transform infrared spectroscopy (FT-IR), have also been used to distinguish different resins. Herein, this paper provides a comprehensive overview of the production process, phytochemistry, traditional uses and quality control of benzoin and looks to the future for promoting its further research and applications.

## 1. Introduction

Plant resin is defined primarily as a lipid-soluble mixture of volatile and nonvolatile terpenoids and/or phenolic secondary compounds. There are two kinds of resins: terpenoid resin and phenolic resin [1]. Benzoin, a representative of phenolic resins, is revealed that mainly contains benzoic acid, cinnamic acid and their derivatives in primary volatile compounds and triterpenoids and lignans in dominant nonvolatile compounds. Several works have stated that crude extractions of benzoin or purified monomers obtained from benzoin exhibit pharmacological activities, such as antitumor, neuroprotective, cytotoxic, antimicrobial, anti-inflammatory and pesticidal properties [2,3,4,5,6]. Furthermore, benzoin can be used as a fragrance in religious rituals along with olibanum; as an industrial material for perfume, detergent and other daily chemicals owing to its distinctive fixation; and as a natural flavoring and natural adjuvant due to its olfactory and preservative features [7].

As members of a group who are interested in exploring the quality differences among a variety of types of commercial benzoin and evaluating techniques for quality assessment, we observe that there are several reviews summarizing the botany, pharmacology and phytochemistry of benzoin. One proposes coniferyl benzoate as a Q-marker (chemicals which can reflect the quality of this Chinese herb) of benzoin due to its prominent bioactivity. However, the process of benzoin’s production has not been reported, which may have an impact on its quality and efficacy. Meanwhile, these reviews summarized compounds identified by GC-MS or isolated from tree bark. We summarize all compounds isolated and identified from the resin only by phytochemistry methods, avoiding incorporating chemicals which are not in benzoin to provide accuracy [8,9]. Third, we give a relatively comprehensive summary of benzoin’s prescriptions for better application in clinical practices. Lastly, a review of the systematic and comprehensive analysis of quality control of benzoin has not been reported. We discuss methods of quality control from four facets to standardize its production and application. To sum up, we aim to conclude a systematic summary concerning the production process, quality control and traditional uses of benzoin as well as all compounds isolated only from benzoin resin, which we expect to provide a useful bibliography for further research and applications of benzoin.

Studies on benzoin published before February 2023 were collected from scientific databases (PubMed, CNKI, Science Direct, Google Scholar and Web of Science), using the keywords “resin”, “benzoin”, “*Styrax*”, “Anxixiang” (Chinese pinyin of benzoin), ”traditional Chinese medicine (TCM)” and combinations thereof.

## 2. The Production of Benzoin

### 2.1. Benzoin-Producing Plants

In order to find out the origin of benzoin, we should understand what benzoin is. It belongs to *Styrax*, the largest genus in the Styracaceae family. *Styrax* is renowned for its resinous material production, which makes it predominant in the family. Benzoin has plentiful names. In Germany, it is called benzoebaum. In Spain, it is bálsamo de Benjuí [9]. It is also categorized into balsam with storax, Tolu balsam and Peru balsam [10]. Some literatures recognize benzoin as benzoin gum, which is confused with the concepts of gum and resin. Gum is a water-soluble chain of polysaccharides [1]. Moreover, two major benzoins universally exist in commercial markets: Siam benzoin and Sumatra benzoin. Although the categorization varies in different places, it is widely shared that Siam benzoin is produced by plants belonging to *S. tonkinensis* (Pierre) Craib ex Hartwiss, while Sumatra benzoin is excreted by trees of *S. benzoin* Dryand and *S. perelleloneurum* Perkins. Some think that Siam benzoin can provide a sweeter and vanilla-like odor, which makes Siam benzoin superior to Sumatra benzoin in the food industry, regardless of prices. In contrast, the Sumatran type is spicier and features a styrax-like odor, playing a role in the daily chemical industry and pharmaceutical preparations [11,12,13]. There are also other benzoin resins produced by *Styrax*, with benzoin in lesser field and quality, such as Bolivia benzoin produced by *S. pearceivar* bolivianum, Bogota Storaque by *S. Aureum* and Solid Storaque by *S. officinale* [14].

Storax and styrax are easily confused, and both have predominant phenolic components and similar olfactory properties. Also, the use of “styrax” to refer to these two resins in the literature worsens this situation. However, the resin produced from the *Styrax* species (Styracaceae) is styrax, while (Altingiaceae) storax is from the *Liquidambar* species [10].

### 2.2. Benzoin-Producing Process

There are a few reports regarding the resin secretory structures of benzoin. Deng et al. revealed that traumatic resin ducts in Styrax are formed by parenchymal cells in secondary xylem by schizolysigeny after injury [15]. The resin is not secreted to the outside of plants until injury take place [1]. Furthermore, continuous injuries could promote the regeneration of new resin ducts [16]. They also discovered that ethephon, a plant growth regulator, can remarkably boost the yield of benzoin by increasing the dimension and number of traumatic resin ducts to promote their distribution [15]. However, the compartmentation sequestration process of benzoin resin has been less researched.

The trees belonging to Styrax have no ability to secrete resin unless their stems get injured. Consequently, benzoin resin is regarded as a pathologic exudation of plants in a stress response to external injuries [17]. Pinyopusarerk described the tapping of benzoin in Laos [18]. The method involves tapping several staggered rectangle notches in the plant’s cambium between 30 centimeters from the ground and the height of the first branches and loosening the bark of the notches’ lower part to induce the formation of benzoin resin. In Malaysia, these notches are inverted triangles, and there are three notches at the same level. In Indonesia, trapezoid-shaped cuts and V-shaped cuts are tapped by wedges between the bark and wood to create incisions and collect benzoin [7]. However, there is no information on the relationship between the size of notch and secretion of benzoin. It should be noted that in dry conditions, the tapped incisions should be cleaned up to ensure the mobility of the resin. However, incisions will be closed by bark to prevent resin exudation in wet years [18]. 

Several weeks after being tapped, the incisions will be covered with benzoin in the form of tears. At first, quite a lot of transparent, soft and viscous gum or oil along with water exudes and flows around the wound. Then, that exudation, exposed to the air, will evaporate and condense into considerably sizable pieces, and become hard, and eventually fragile resin forms. In that process, evaporation of volatile components, oxidation and polymerization take place. The chemical composition of the resin becomes more complex, and the pale fresh benzoin darkens, so that it exhibits similar features and characteristics to benzoin. It is shown that trees with dark, thick and rough bark can produce more benzoin. Also, a tree in a larger diameter class produces more benzoin [7,19].

Farmers can collect benzoin several times from one tree. The first resin flow is called takasan, with white inner resin and yellowish resin. The second resin flow is lecet, whose color ranges from white to dark brown. The third resin flow is named tahir or juror. Differing from frankincense, with which more valuable resins occur in its second or third woundings, the first flow of benzoin resin has fewer impurities and dries more easily [7].

## 3. Phytochemistry

To better study the characteristics of benzoin, we should remember that the chemical compounds in benzoin show distinctiveness from other traditional Chinese herbs, even those from the same category of resin Chinese medicines. Based on previous studies, benzoin is composed of various chemical compounds, including balsamic acid esters, lignans, terpenoids and sesquiterpene. In addition, cinnamic acid, benzoic acid and their derivatives, the most abundant constituents in benzoin, play an indispensable role in bioactivity. Other reviews have focused on chemical components identified by gas chromatography mass spectrometry (GC-MS) or isolated from the stem bark of plants secreting benzoin [8,9]. Herein, we comprehensively summarize the compounds of benzoin resin obtained from phytochemical isolation, which are shown in Table 1.

### 3.1. Balsamic Acid Esters

**Figure 1:**
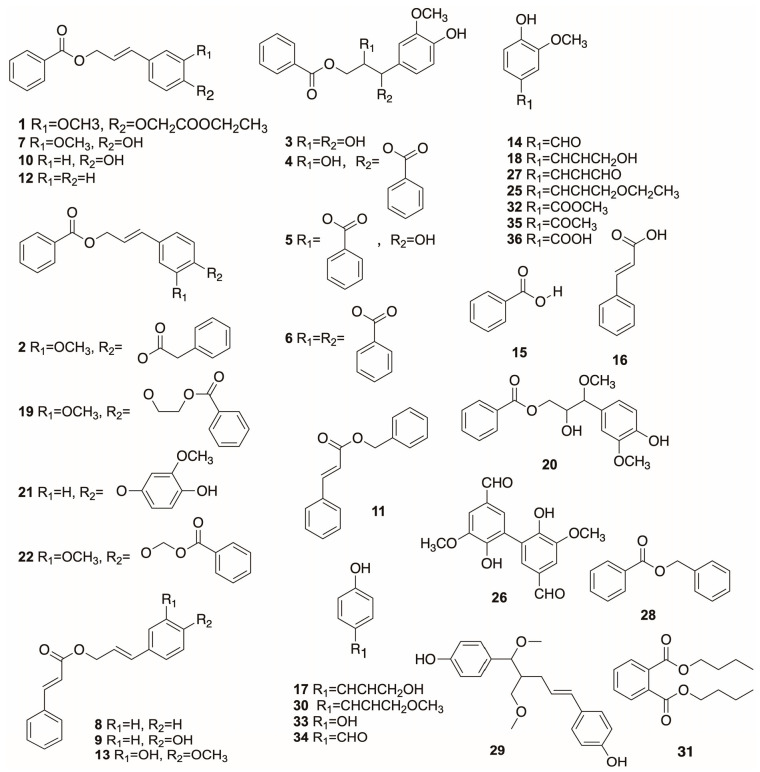
The structures of balsamic acid esters obtained from benzoin resin.

Balsamic acid esters are the most crucial constituents in benzoin. There are 37 balsamic acid esters isolated from benzoin (Figure 1), among which benzoic acid, cinnamate acid and their esters have predominant content in benzoin [20,21]. In addition, one of the major differences between *S. benzoin* Dryand, *S. paralleloneurum* and *S. tonkinensis* is that the first two benzoins contain a large amount of cinnamic acid and its esters, while benzoic acid and its esters predominate in *S. tonkinensis* [12]. As a result, the content of balsamic acids is represented by cinnamic acid in *S. officinalis* and *S. paralleloneurum*, while benzoic acid is in *S. tonkinensis* [22].

### 3.2. Lignans

**Figure 2:**
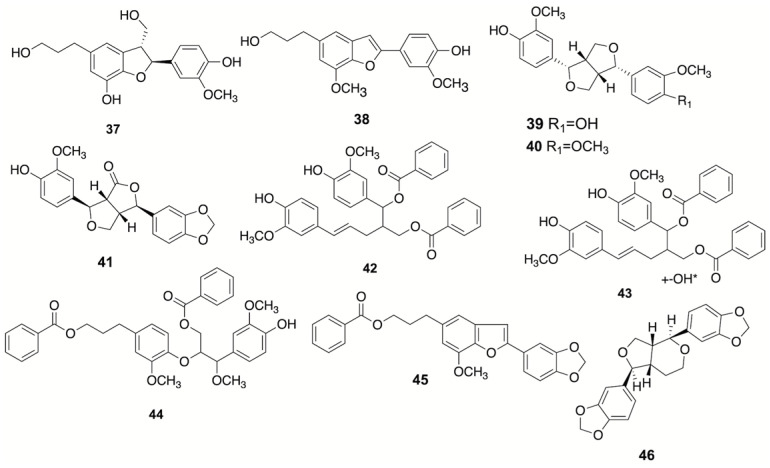
The structures of lignans obtained from benzoin resin.

There are eight lignans isolated from benzoin (Figure 2), including benzofuran lignans, tetrahydrofuranoid lignans, neolignane and oxyneolignan [4,6,23,24,25].

### 3.3. Terpenoids

**Figure 3:**
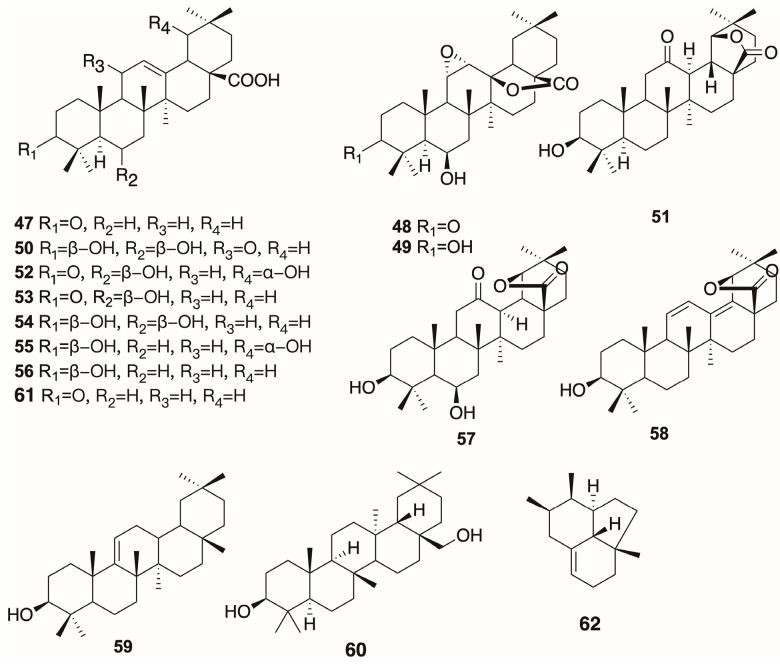
The structures of terpenoids obtained from benzoin resin.

There are 15 pentacyclic triterpenoids (Figure 3), which are all oleanane-type triterpenoids, as well as only one special sesquiterpenoid discovered in benzoin [4,24,26,27,28,29]. The bioactivities of their cytotoxic and differentiation-inducing effects demonstrate their potential for anticancer treatments [29,30].

**Table 1:** Chemical compounds isolated from benzoin resin.

No.	Compounds	Type	Ref.
1	(*E*)-3-[3-methoxy-4-(2-ethoxy-2-oxoethoxy)-phenyl] allyl benzoate	Balsamic acid esters	[23]
2	stybenpropol A	Balsamic acid esters	[6]
3	Siamyl benzoate	Balsamic acid ester	[24]
4	Siamyl-1,3-dibenzoate	Balsamic acid ester	[24]
5	Siamyl-1,2-dibenzoate	Balsamic acid ester	[24]
6	Siamyl-2,3-dibenzoate	Balsamic acid ester	[24]
7	coniferyl benzoate	Balsamic acid ester	[24]
8	cinnamyl cinnamate	Balsamic acid ester	[24]
9	*p*-coumaryl cinnamate	Balsamic acid ester	[24]
10	*p*-coumaryl benzoate	Balsamic acid esters	[24]
11	benzyl cinnamate	Balsamic acid esters	[24]
12	cinnamyl benzoate	Balsamic acid esters	[24]
13	ciniferyl cinnamate	Balsamic acid esters	[24]
14	Vanillin	Balsamic acid esters	[24]
15	benzoic acid	Balsamic acid esters	[24]
16	cinnamic acid	Balsamic acid esters	[24]
17	*p*-coumaryl alcohol	Balsamic acid esters	[20]
18	coniferyl alcohol	Balsamic acid esters	[31]
19	tonkinensisin B	Balsamic acid esters	[25]
20	tonkinensisin C	Balsamic acid esters	[25]
21	stytonkinol A	Balsamic acid esters	[32]
22	stytonkinol B	Balsamic acid esters	[32]
23	*trans*-(tetrahydro-2-(4-hydroxy-3-methoxyphenyl)-5-oxofuran-3-yl) methyl benzoate	Balsamic acid esters	[2]
24	3-(4-hydroxy-3-methoxyphenyl)-2-oxopropylbenzoate	Balsamic acid esters	[2]
25	4-((*E*)-3-ethoxypropl-1-enyl)-2-methoxyphenol	Balsamic acid esters	[2]
26	Dehydrodivanillin	Balsamic acid esters	[2]
27	coniferyl aldehyde	Balsamic acid esters	[2]
28	benzyl benzoate	Balsamic acid esters	[26]
29	(4E)-1,5-bis(4-hydroxyphenyl)-1-methoxy-2-(methoxymethyl)-4-pentene	Balsamic acid esters	[4]
30	(E)-p-coumaryl alcohol-γ-O-methyl ether	Balsamic acid esters	[4]
31	dibutyl phthalate	Balsamic acid esters	[4]
32	methyl 4-hydroxy-3-methoxybenzoate	Balsamic acid esters	[4]
33	p-hydroxybenzaldehyde	Balsamic acid esters	[4]
34	p-hydroxyacetophenone	Balsamic acid esters	[4]
35	Acetovanillone	Balsamic acid esters	[4]
37	4-hydroxy-3-methoxy-benzoic acid	Balsamic acid esters	[4]
37	(+)-cedrus	Lignan (benzofuran lignan)	[23]
38	2-(4-hydroxy-3-methoxyphenyl)-5-(3-hydroxypropyl)-7-methoxybenzofuran	Lignan (benzofuran lignan)	[6]
39	(+)-pinoresinol	Lignan (tetrahydrofuranoid lignan)	[6]
40	(+)-pinoresinol monomethy ether	Lignan (tetrahydrofuranoid lignan)	[6]
41	styraxin	Lignan (tetrahydrofuranoid lignan)	[6]
42	siam morinol	Lignan (neolignane)	[24]
43	siam hydroxymorinol	Lignan (neolignane)	[24]
44	tonkinensisin A	Lignan (oxyneolignan)	[25]
45	5-(3’’-benzoyl-oxypropyl)-7-methoxy-2-(3’,4’-methylenedioxy phenyl) benzofuran	Lignan (benzofuran lignan)	[4]
46	Sesamin	Lignan (tetrahydrofuranoid lignan)	[4]
47	3-oxo-siaresinolic acid	Triterpenoid	[24]
48	6β-hydroxy-3-oxo-11α,12α-epoxyolean-28,13β-olide	Triterpenoid	[29]
49	3β,6β-dihydroxy-11α,12α-epoxyolean-28,13β-olide	Triterpenoid	[29]
50	3β,6β-dihydroxy-11-oxo-olean-12-en-28-oic acid	Triterpenoid	[29]
51	3β-hydroxy12-oxo-13Hα-olean-28,19β-olide	Triterpenoid	[29]
52	19α-hydroxy-3-oxo-olean-12-en-28-oic acid	Triterpenoid	[29]
53	6β-hydroxyl-3-oxo-olean-12-en-28-oic acid	Triterpenoid	[29]
54	sumaresinolic acid	Triterpenoid	[29]
55	siaresinolic acid	Triterpenoid	[29]
56	oleanolic acid	Triterpenoid	[29]
57	3β,6β-dihydroxy-12-oxo-13Hα-olean-28,19β-olide	Triterpenoid	[27]
58	3-oxo-olean-11,13-dien-28,19β-olide	Triterpenoid	[27]
59	Myrsinene	Triterpenoid	[26]
60	Myricadiol	Triterpenoid	[4]
61	3-keto-oleanonic acid	Triterpenoid	[4]
62	(−)-1,2,2α,3,3,4,6,7,8,8a-decahydro-2α,7,8-trimethyl acenaphthylene	Sesquiterpenoid	[28]

## 4. Traditional Uses

### 4.1. Non-Medicinal Use

Benzoin is used in various religious ritual ceremonies. It is known that benzoin is acrid, exceedingly sweet-scented and has a strong vanilla smell. The smoke produced from burning benzoin was said in ancient Malayan to eliminate the negative impact of evil spirits and diseases [9]. The use of benzoin in China was first included in The Book of Jin, which was as a type of incense [33]. In the Compendium of Materia Medica, benzoin was apotropaic [34]. In Sumatra, people use benzoin burnt with coffee, obtaining a vanilla-like fragrance to relax [11]. Besides contributing its own fragrance, its tenacity and ability to prevent the loss of middle and top notes of more volatile compounds makes it popular in industries such as perfumes, cosmetics, soap and detergents [7]. Its prominent antioxidant ability also contributes to its application in the cosmetics industry [35]. However, the use of benzoin may decrease in religious ceremonies because it is considered outdated and also in industry due to the use of synthetic flavoring materials.

Benzoin can be used as a flavor enhancer in the food industry. Benzoin can produce a vanilla-like odor due to the presence of vanillin and a chocolate-like flavor due to the existence of cinnamates [7]. Benzoin is mixed with tobacco in Klembak menyan, a kind of cigarette produced in central Java [36]. Romans and Greeks also put benzoin in their traditional dishes [11]. In the United States, benzoin was approved for food use in 1992. Since the latest Code of Federal regulation, benzoin has been allowed to be used in food as a natural flavoring substance and natural adjuvant. Benzoic acid, a notable preservative used in food, beverages and pharmaceutical preparations, was originally separated from benzoin resin. Benzoin can also be used as a glazing agent, and benzoin tincture is employed in chocolate eggs to increase luster. In Japan, benzoin is used as an ingredient in chewing gum [7].

### 4.2. Medicinal Use

ChP2020 records that benzoin has efficacy in inducing resuscitation, restoring consciousness, dispersing Qi, activating blood circulation and relieving pain, with indications for chest pains, infantile convulsions and unconsciousness resulting from stroke [37]. According to modern pharmacological research, benzoin has antimicrobial, anti-inflammatory, pesticidal, antitumor, neuroprotective activities, etc., which has been fully introduced in other articles. As an important traditional Chinese medicine used throughout history, benzoin has been applied in 95 Chinese herbal prescriptions for treating various diseases [38]. The earliest recorded medicinal history of benzoin was in the Tang materia medica, in which it was used to treat diseases caused by phlegm-dampness accumulation in the chest and abdomen, resulting in symptoms such as palpitations, chest tightness and difficulty breathing [39]. In the Compendium of Materia Medica, it is recorded that benzoin can be used to treat a variety of diseases, such as conditions resulting by the death of a fetus, symptoms of being bitten or poisoned by poison, cholera and rheumatism, and symptoms that occur during menstruation and postpartum recovery. It can also alleviate some mental problems, such as nightmares, insomnia and exhaustion resulting from the diseases [34]. Commonly used TCM prescriptions containing benzoin are listed in Table 2. Herein, we summarize several common medical uses of benzoin. Benzoin has two kinds of tincture preparations. Compound tincture of benzoin consists of benzoin, styrax, balsam of tolu and aloe in alcohol, whereas tincture of benzoin is composed of 10% benzoin in alcohol [40]. Tinctures of benzoin are allergenic on contact, but there are few reports about this. Their antimicrobial and disinfectant activities make them useful in the treatment of cough, laryngitis, bronchitis and other respiratory disorders. They can mildly stimulate respiratory mucosa, increase tracheal and bronchial secretions, and dilute sputum to be discharged more easily, so as to achieve the purpose of expectoration and coughing. They can be used to prevent blister formation and nipple cracking, as local skin antibiotics to promote wound healing, as an adhesive to increase the tackiness of dressings, as a surgical tape for dermatologic and plastic surgery, and as an antiseptic. The compound Podophyllum Paint, consisting of podopgyllum resin and compound benzoin tincture, is used to treat warts.

## 5. Quality Control

### 5.1. Inspection

Quality control is paramount for Chinese herbs to ensure their efficacy, which is a standard to monitor their quality. The criteria of benzoin from Chinese pharmacopoeia (ChP 2020), USA pharmacopoeia (USP43-NF38), European pharmacopoeia (EP10.0), Japanese pharmacopoeia (JP18) and Indian pharmacopoeia (IP2018) are summarized in Table 3, in which content determination, ethanol-insoluble matter, acid-insoluble ash, loss on drying, total ash and presence of dammar gum require detailed analysis [22,37,44,45,46]. Dammar gum is often adulterated in a semi-processed form of block benzoin, once resulting in a serious drop in benzoin’s price [47,48]. In addition, organoleptic properties, such as the color, odor and taste of benzoin resin, also need to be considered. Beyond that, quite a few researchers also point out that the quality of benzoin is related to its size and purity. The larger and purer the resin tear, the higher the value [18]. It is thought that fine crushed benzoin dust, which offers a greater surficial area, would facilitate oxidation and the loss of vital volatiles, and furthermore, impair its olfactory properties. In addition, due to viscidity, bark and other extraneous materials, such as sand and dirt, are easily mixed into benzoin during the process of collection and storage [7].

### 5.2. Content Determination

In addition, as a strong analytic method, high-performance liquid chromatography (HPLC) has been established to achieve the quantitative quality control of benzoin, determining the content of benzoic acid, cinnamic acid and coniferyl benzoate separately, and vanillin, coniferyl aldehyde and trans-[tetrahydro-2-(4-hydroxy-3-methoxyphenyl)-5-oxofuran-3-yl] methyl benzoate simultaneously [49,50,51,52]. Besides this, UV-Vis spectrophotometry has been developed to determine the content of total triterpenoids in benzoin [53].

In addition, Xie et al. established a method of determining the content of benzoic acid and coniferyl aldehyde simultaneously by HPLC, and the color of benzoin (surface and cross-sectional) by spectrophotometer, drawing the conclusion that when the surface is yellowish white and the fractured surface is ivory white, benzoin has a higher content of benzoic acid and coniferyl benzoate, which is consistent with the color requirements in ChP2020 [54].

### 5.3. Qualitative Analysis

It is universally acknowledged that the pharmacological effects of traditional Chinese herbs in clinical practice are generally the sum of the effects of various chemicals, which interact with each other. Given these factors, the analysis of only some compounds is insufficient for integrated quality control. Furthermore, compounds of benzoin are quite variable and differ in original plants and their geographic conditions, methods of collection and the process of extraction [55]. Therefore, GC-MS is introduced to optimize the rapid identification of the multiple chemicals in benzoin so as to provide an instrument for the quality control and supervision of benzoin.

**Table 3:** The regulations of benzoin in various countries and regions.

Inspection	ChP 2020	EP10.0	JP18	IP2018	USP43-NF38
Benzoin type	*S. tonkinensis* (Pierre) Craib ex Hart	*S. benzoin* Dryander as Sumatra benzoin; S. tonkinensis (Pierre) Craib ex Hart as Siam benzoin	*S. benzoin* Dryander or other species of Styracaceae	*S. benzoin* Dryand and *S. paralleloneurus* Perkins as Sumatra benzoin, *S. tonkinensis* (Pierre) Craib ex Hart and other species of the Section Anthostyrax of the genus Styrax as Siam benzoin	*S. benzoin* Dryand and *S. paralleloneurus* Perkins as Sumatra benzoin, *S. tonkinensis* (Pierre) Craib ex Hart and other species of the Section Anthostyrax of the genus Styrax as Siam benzoin
Content determination	Total balsamic acid, calculated as benzoic acid, which should not be less than 27.0% of resin	Benzoic acid should be 35.0% to 55.0% of total acids in Siam benzoin and 25.9% to 50.0% in Sumatra benzoin	-	Total balsamic acid is determined, which should not be less than 25.0% of dried resin, calculated as cinnamic acid in Sumatra benzoin and as benzoic acid in Siam benzoin	Benzoic acid is determined by the weight of the residue remining after the reaction of benzoin with warm carbon disulphide; the residue is 6.0% of benzoin in Sumatra benzoin and 12.0% in Siam benzoin
Ethanol-insoluble matter	Max 2.0%	Max 5.0 % in Siam benzoin; max 20.0 % in Siam benzoin	Max 30.0%	Max 25.0% in Sumatra benzoin; max 10.0% in Siam benzoin	Max 10.0% in Siam benzoin; max 25.0% in Siam benzoin
Acid-insoluble ash	-	Max 2.0 %	Max 1.0%	Max 1.0% in Sumatra benzoin; max 0.5 % in Siam benzoin	Max 1.0% in Sumatra benzoin; max 0.5% in Siam benzoin
Loss on drying	Max 2.0%	Max 5.0 %	-	Max 10.0%	-
Total ash	Max 0.5%	Max 2.0 %	Max 2.0%	-	-
Presence of dammar gum	-	Thin-layer chromatography	-	Thin-layer chromatography	-

The combination of the excellent separation ability of GC with the strong detection and identification capacity of MS and the availability of a large amount of commercial and “in-house” chemical databases makes it widely used in the analysis of volatiles and semi-volatiles. GC-MS for analyzing benzoin widely combines alkaline hydrolysis and derivation of polar groups, such as methylation and diazomethane. Pastorova applied derivatization with N, O-bis(trimethylsily)trifluroaccetamide and trimethychlorosilane so as to determine aromatic acids and alcohol building blocks as well as their esters in different combinations in one analytical method [55].

Lou et al. showed that differing from other volatile oils which mainly contain terpenoids, benzoin volatile oil has more aromatic compounds, including benzyl cinnamate, cinnamyl cinnamate, 3-phenyl-2-propenal, styrene, benzaldehyde and benzyl benzoate, consistent with the above conclusion: balsamic acid esters are the predominant compounds in benzoin [56]. Peng et al. compared the impact of extraction methods on the obtained compounds, which revealed that steam distillation could obtain more volatile compounds and headspace solid-phase microextraction (HS-SPME) could detect more low-polarity compounds [57]. However, Fernandez et al. also indicated that a slice of low-volatile components, such as cinnamate, cinnamyl benzoate and cinnamyl cinnamate, could not be identified by SPME. Meanwhile, some new components, ethanol, toluene, α- and β-pinene, linalool oxide, terpinene-4-ol, α-analgene and other sesquiterpenes, could be identified by SPME due to the fiber selectivity and low volatility of benzyl benzoate [35]. The compounds detected in crushed benzoin and the essential oil are also different. Castel et al. showed that the volatile chemicals in crushed benzoin mainly contained benzaldehyde, 𝛂-pinene, benzyl alcohol, methyl benzoate, benzoic acid and vanillin. However, the dominant components in the essential oil were benzyl benzoate, allyl benzoate, methyl benzoate, benzaldehyde and R-pinene [58]. Fernandez et al. also indicated that some new compounds, especially benzyl alcohol and vanillin, are found only in crushed benzoin but not in the essential oil. They further draw a comparison between volatile extracts in the essential oils of Sumatra benzoin and Siam benzoin, which demonstrated that there was aromatic carboxylic acid, esters and sesquiterpene hydrocarbons in both benzoin oils, while major components were benzoic acid, methyl benzoate and allyl benzoate in Siam benzoin, and styrene, cinnamic acid and benzyl cinnamate in Sumatra benzoin [35].

### 5.4. Species Identification

Although benzoin resin has a long history of application, the identification of benzoin resin is not yet totally accurate. Given its special biological basis, it substantially differs from botanical drugs, which adds the difficulties of species identification. In addition, the adulteration of different resins makes the situation worse. In the meantime, the different uses of the two resins, where Siam benzoin is mainly used for incense while Sumatra benzoin is used in pharmaceutical industries and in varnishing wood, make species identification more indispensable. DNA molecular biology is widely used in species and adulterant identification. However, it cannot be used for benzoin, given the peculiarities of resin. EP10.0 adopts morphological examination and thin-layer chromatography to distinguish Siam and Sumatra benzoin [45]. Whereas the former is based on the experience of practitioners and influenced by multitudinous factors, the latter is time-consuming and inefficient. The method reported by Fernandez et al. dramatically deals with these problems [59]. They identify species of benzoin and potential counterfeited benzoin based on its olfactive features by electronic nose technology. Most of the samples had a recognition score above 90%, indicating significant difference between Sumatra and Siam benzoins in term of olfactive features. Fourier-transform infrared spectroscopy (FT-IR) spectra were also used to separate the different species of benzoin. Differing from the content and infrared absorption bands of benzoic acid and cinnamic acid, Sumatra benzoin and Siam benzoin have subtle differences in 1000 cm^−1^ to 600 cm^−1^, which could be a significant basis of benzoin species identification [60]. Hovaneissian et al. used HPLC-PAD-fluorimetry to separate Siam and Sumatra benzoin, considering coniferyl benzoate as a fluorescence marker of Siam benzoin, while no compounds in Sumatra benzoin showed the same fluorescence [17].

The non-volatile fraction of benzoin has seldomly been analyzed. However, Courel et al. considered pentacyclic triterpenoids as markers to identify various balsams and detect counterfeited balsams. Due to their high specificity, low volatility and stability, triteropenes prior to aromatic compounds in the facet of unambiguous identification of archaeological balsams [10].

Furthermore, methods of distinguishing different resins have been developed simultaneously. Taiti et al. used proton transfer reaction time-of-flight mass spectrometry (PTR-ToF-MS) to classify different resins based on their volatile organic compound emission profiles [61]. Wei et al. established a rapid and nondestructive method for identification of six resins by near-infrared reflectance spectroscopy (NIR). In this study, researchers applied principal component analysis (PCA) to NIR data, and six resin herbs (benzoin, succinum, myrrha, olibanum, colophonium and bambusae concretio silicea) were discriminated using support vector machine (SVM) algorithms, the predicting accuracy of which is up to 100% [62].

On the other hand, benzoin in China is completely based on imports, bringing huge difficulties in quality supervision. Seeking alternatives in China based on plant affinity is an effective measure. As an example, in 1964, Lian et al. discovered that according to qualitative and quantitative analysis, S. hypogiaucus Perk, S. mrothysus Perk and S. subniveus Merr. et Chun can be perceived as alternatives of medical benzoin [63]. Subsequent works should attach more importance to their pharmacological activities and the possibilities of further utilization and production.

## 6. Conclusions and Perspectives

This review has summarized the research progress of the production, phytochemistry, quality control and traditional use of benzoin, providing comprehensive information on this resin. Based on previous records and research, benzoin has a wide range of biological and pharmacological activities and has been widely used in a host of fields for a long time. Heretofore, a total of 62 compounds have been isolated and identified in this resin, including balsamic acid esters, lignans and terpenoids. However, the bioactivities and action mechanisms of these isolated compounds have not been fully discussed. Systematic efficacy studies are imperative to identify the bioactive compounds responsible for their pharmacological effects.

On the other hand, although benzoin has a long history of application in incense, food, daily chemical and pharmaceutical industries, systematic evaluations of its safety and toxicity are still limited, which should be resolved and verified by in-depth research and clinical trials. In addition, the efficacy of benzoin is frequently achieved by working with other TCMs. Nevertheless, the action mechanisms of those prescriptions have not been comprehensively elaborated. For better application in clinical practices, the action mechanism, material basis and relationship between them should be improved when considering these prescriptions.

In terms of quality control, the current quality criteria are only limited to benzoic acid or cinnamate acid, which cannot achieve an integrated evaluation of the quality of benzoin. Therefore, developing more chemical and bioactive markers which are related to its quality is indispensable. In addition, chemicals in benzoin vary in collection stage, origin plants, and geographical and climatic conditions. Establishing a standard quality method concerning those influencing factors should also be taken into consideration.

## Figures and Tables

**Table 2:** The traditional and clinical uses of benzoin in China.

Prescription Name	Main Compositions	Traditional and Clinical Uses	Ref.
Suhe Pill	Styrax, Caryoohylli Flos, Benzoin, Olibanum, Aucklandiae Radix, Santali Albi Lignum, Anisi Stellati Fructus, Cyperi Rhizoma, Atractylodis Macrocephalae Rhizoma, Chebulae Fructus, Piperis Longi Fructus, Cinnabaris, Borneolum	Stroke, coma, vomiting and diarrhea, abdominal pain, convulsive phlegm, infantile convulsions	[41]
Tongluo Huoxue Pills	Bovis Calculus Artifactus, Cyperi Rhizoma, Paeoniae Radix Rubra, Chuanxiong Rhizoma, Borneolum, Squama Manitis, Carapax Et Plastrum Testudinis, Notopterygii Rhizoma Et Radix, Draconis Sanguis, Gastrodiae Rhizoma, Rosin, Three Snack, Clematidis Radix Rhizoma, Cinnamomi Cortex, Benzoin, Scorpio, Myrrha, Carthami Flos, Ephedrae Herba, Alpiniae Katsumadai Semen, Chrysophoron, Asari Radix Et Rhizoma, Glycyrrhizae Radix Et Rhizoma, Angelicae Sinensis Radix, Curcumae Longae Rhizoma	Cerebrovascular accident sequelae	[42]
Qingpeng Plaster	Oxytropis Falcate Bunge, Arhei Pumili, Tie Bang Tui, Chebulae Fructus, Terminaliae Belliricae Fructus, Phyllanthi Fructus, Benzoin, Tinospora Sinensis, Moschus	Gout, rheumatism, rheumatoid arthritis, itch in skin, eczema	[43]
Tongqiao Zhentong Powder	Acori Tatarinowii Rhizoma, Curcumae Radix, Piperis Longi Fructus, Cyperi Rhizoma, Aucklandiae Radix, Caryophylli Flos, Santali Albi Lignum, Aquilariae Lignum Resinatum, Styrax, Benzoin, Olibanum, Borneolum	Cardiothoracic pain, cholera, vomiting and diarrhea	[37]
Yixin Wan	Ginseng Radix Et Rhizoma Rubra, Bubali Cornu, Bufonis Venenum, Borneolum, Carthami Flos, Bovis Calculus Artifactus, Heishunpian, Notoginseng Radix Et Rhizoma, Benozin, Margarita	Angina pectoris and coronary heart disease	[37]

## Data Availability

Not applicable.
